# Deaf Adults’ Health Literacy and Access to Health Information: Protocol for a Multicenter Mixed Methods Study

**DOI:** 10.2196/14889

**Published:** 2019-10-09

**Authors:** Michael M McKee, Peter C Hauser, Sara Champlin, Michael Paasche-Orlow, Kelley Wyse, Jessica Cuculick, Lorraine R Buis, Melissa Plegue, Ananda Sen, Michael D Fetters

**Affiliations:** 1 Department of Family Medicine University of Michigan Ann Arbor, MI United States; 2 Department of American Sign Language and Interpreter Education Rochester Institute of Technology Rochester, NY United States; 3 Mayborn School of Journalism University of North Texas Denton, TX United States; 4 Section of General Internal Medicine Boston University Boston, MA United States; 5 Center on Cognition and Language National Technical Institute for Deaf Rochester Institute of Technology Rochester, NY United States

**Keywords:** deaf, hearing loss, consumer health information, health literacy

## Abstract

**Background:**

Deaf American Sign Language (ASL) users often struggle with limited health literacy compared with their hearing peers. However, the mechanisms driving limited health literacy and how this may impact access to and understanding of health information for Deaf individuals have not been determined. Deaf individuals are more likely than hearing individuals to use the internet, yet they continue to report significant barriers to health information. This study presents an opportunity to identify key targets that impact information access for a largely marginalized population.

**Objective:**

This study aims to elucidate the role of information marginalization on health literacy in Deaf ASL users and to better understand the mechanisms of health literacy in this population for the purpose of identifying viable targets for future health literacy interventions.

**Methods:**

This is an exploratory mixed methods study to identify predictors and moderators of health literacy in the Deaf population. These predictors of health literacy will be used to inform the second step that qualitatively explains the findings, including how Deaf individuals access and understand Web-based health information. Multiple interviewer- and computer-based instruments underwent translation and adaptation, from English to ASL, to make them accessible for the Deaf participants in our study. A planned sample of 450 Deaf ASL users and 450 hearing native English speakers, aged 18 to 70 years, will be recruited from 3 partnering sites: Rochester, NY; Flint, MI; and Chicago, IL. These individuals will participate in a single data collection visit. A subset of participants (approximately 30) with key characteristics of interest will be invited for a second data collection visit to observe and inquire more about their ability to directly access, navigate, and comprehend Web-based health information. The study will help assess how the ways health literacy and information are visualized may differ between Deaf individuals and hearing individuals. The study will also survey participants’ ownership and use of computer and mobile devices and their level of Web-based information use, including health information.

**Results:**

Adaptation and translation of protocols and instruments have been completed and are now in use for the study. Recruitment is underway and will continue until late 2020. Results from this study will be used to provide a guide on how to structure Web-based health information in a way that maximizes accessibility and improves health literacy for Deaf individuals.

**Conclusions:**

The results from this mixed methods proposal will advance what is known about health literacy and health information accessibility for Deaf individuals. This innovative study will generate rich data on how to formulate health information and health literacy interventions more accurately to take advantage of visual learning skills.

**International Registered Report Identifier (IRRID):**

PRR1-10.2196/14889

## Introduction

### Background

Deaf American Sign Language (ASL) users are nearly 7 times more likely than their hearing peers to have inadequate health literacy [1]. Deaf ASL users (henceforth, Deaf) rarely receive language concordant health care services and are at highest risk for miscommunication with their health care providers. Deaf individuals understand less than 30% of what is being said through lipreading [2,3]. Furthermore, prose literacy poses a challenge for Deaf individuals; the average Deaf individual reads English at the fifth- to sixth-grade level [1,4-7]. The use of interpreters is the standard of care for Deaf ASL users, yet they are infrequently provided [8]. The majority of Deaf individuals (approximately 95%) have hearing family members who do not sign. Thus, many experience the *dinner table* syndrome, where they have encountered years at the dinner table watching close family members and friends converse with each other while being unable to understand what is being said, depriving them of incidental learning opportunities that many hearing individuals take for granted [9]. The loss of incidental learning opportunities and information marginalization for Deaf individuals occurs daily in a broad range of work, schools, friends, families, government, media, and health care contexts [1]. Many Deaf ASL users learn language, health information, and even culture via peers rather than family [10] and struggle to identify and correct misinformation [11]. Due to this communication-depleted milieu, inadequate health literacy may be an important cause of the lower level of health-related knowledge and worse health outcomes that have been observed among Deaf individuals.

Deaf individuals, because of their social and language marginalization, appear to use Web-based health information more frequently than the general population [12,13]. However, it is unclear if Deaf individuals access Web-based health information or other information sources effectively and what degree of impact this may have on their health literacy. In the general population, there is evidence that health literacy impacts mortality, yet the connection remains inconsistent when looking at other clinical outcomes [14]. In multiple studies, positive associations with both health literacy and electronic health (eHealth) literacy and improved quality of life, better use of health care services, and improved health behaviors among individuals with different types of health conditions were demonstrated [15-17]. An area of concern is that disadvantaged populations, notably those with chronic health conditions or disabilities, may struggle with eHealth literacy. This may promote additional inequalities in their ability for Web-based health information to influence positive outcomes such as self-management of health care needs in these individuals. What is needed is to understand how to design technology and Web-based health information in a way that may benefit these types of consumers [18,19]. As Deaf individuals communicate through a visual language, the proposed study provides an opportunity to determine optimal visual-based information sources, providing another avenue to help those with lower health literacy. Individuals with low health literacy struggle in locating relevant Web-based health information and may fixate on irrelevant aspects of displayed information [20]. Such a phenomenon may be important for populations such as the Deaf who are dependent on visual mechanisms for communication and for overcoming information gaps.

This study describes the methods used to study the role of information marginalization on health literacy for Deaf individuals. The authors describe approaches for instrument adaptations and measures applicable for Deaf individuals who use ASL. Finally, a description of the unique aspects of our proposed data collection method is shared in this paper. The research objectives are two-fold: (1) to elucidate the role of information marginalization on health literacy in Deaf signers and (2) to better understand the mechanisms of health literacy in this population for the purpose of identifying viable targets for future health literacy intervention development.

### Study’s Theoretical Framework

**Figure 1:**
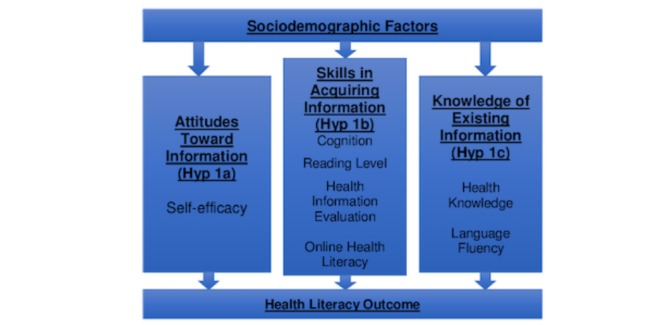
Deaf health literacy conceptual model for information.

There are few mechanism-based studies in health literacy research and none involving Deaf and hard of hearing individuals. A review of the literature helped with developing ideal conceptual models to guide the project’s proposal. The authors modified and created a hybrid of 3 widely used health literacy and diffusion theory conceptual models: (1) the attitudes, skills, and knowledge (ASK) model [21], (2) the health literacy model [22], and (3) the diffusion theory [23] for Deaf individuals. Figure 1 illustrates constructs that are relevant to health literacy and health-related information. Sociodemographics, cognition, educational achievement including reading skills and task performances, and general health knowledge have all been shown to be related to each other with respect to health literacy in the general population [21]. Additional aspects of health literacy that need to be better understood in the Deaf community include the role of attitudes (ie, the feeling and trust one has toward information), skills (ie, the tools that allow one to seek, obtain, and understand information), and knowledge (ie, demonstrates comprehension). It is hypothesized that the factors outlined in the model are key elements of health literacy for Deaf individuals. As a result, our Deaf Health Literacy conceptual model regarding information includes 3 main concepts that lead to health outcomes and are being used in this study to inform data collection and analysis.

To help explain how and why certain characteristics may impact health literacy, including ability to access, navigate, and understand Web-based health information, a qualitative interview along with thematic analysis will be performed. This will explain the interrelation in outcomes of interest (eg, what characteristics are associated with adequate health literacy), intermediary outcomes (eg, what backgrounds, skills, and contextual factors allow for better navigational ability on the Web), and personal conditions that need to be explored further (eg, cognitive issues).

## Methods

### Overview

For this study, there are 3 proposed aims. Aim 1 is to evaluate differences in ASK, regarding health information for both Deaf signers and hearing English speakers. Aim 2 plans to assess hearing status as an effect modifier of the association between health literacy and ASK with health information. Aim 3 will qualitatively assess the impact of health information accessibility and patterns of use on health literacy with one-on-one elicitation interviews. This will be based on actual Web-based health information searches for 4 clinical vignettes using an observed task fulfillment experiment as a prompt. The goal of this aim is to explore differences with Web-based health information search ability and health knowledge acquisition in a subsample of Deaf and hearing individuals recruited from aims 1 and 2. The University of Michigan institutional review board (IRB) approved the study and provides coordination with the following IRBs: Sinai Hospital, Rochester Insitute of Technology, and Hurley Medical Center (HMC). The study is also registered at the ClinicalTrials.gov under ID NCT03093779.

### Study Design

**Figure 2:**
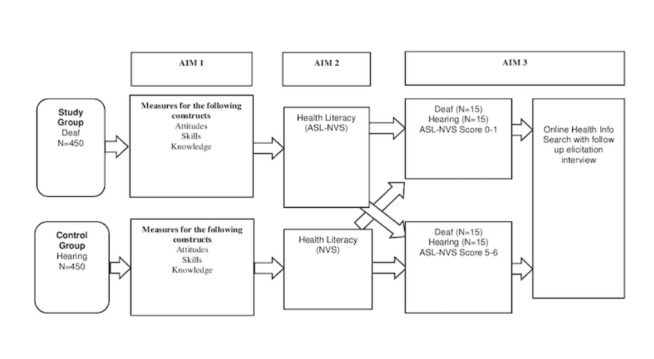
Overall study design. ASL-NVS: American sign language-newest vital sign.

The study uses an explanatory sequential mixed methods design [24] with extensive quantitative data collection procedures to identify predictors and moderators of health literacy. We will first conduct a cross-sectional study to collect predictors of health literacy. These predictors of health literacy will be used to inform the subsequent phase of qualitative assessment. For example, elicitation interviews with selected participants returning for the second data collection visit will be used to help explain the quantitative results and to help elucidate how Deaf individuals access and understand Web-based health information. The study will use a stratified sampling approach to invite participants of certain characteristics to return for further data collection. Characteristics of interest will be based on quantitative analyses that show significant associations between health literacy and any of the measured variables (eg, educational attainment, age, or reading literacy). Approximately 15 Deaf and 15 hearing participants with inadequate health literacy and another 15 Deaf and 15 hearing participants with adequate health literacy will return for a second visit to conduct a task performance on a desktop computer and participate in a follow-up interview designed to learn more about their decision making and preferences. The return visit will involve a qualitative assessment using elicitation interviews to help explain any of the quantitative results. The goal is to learn how and why Deaf individuals access and understand health information (Figure 2) and how this may differ from hearing individuals.

The project uses a community advisory board (CAB) with representation from study sites. The CAB is designed to provide community oversight and guidance on the research protocol development and interpretation of findings. The board consists of 6 Deaf community members, 2 from each of the research data collection sites.

### Study Setting and Participants

The study will recruit 450 Deaf and 450 hearing participants at 3 primary data collection sites. The sites include HMC at Flint, MI (selected because of its diverse Deaf patient population, along with previous research collaborations with the University of Michigan); The National Technical Institute of the Deaf (NTID), which is housed at the Rochester Institute of Technology in Rochester, NY (selected because of NTID’s experience in research and Rochester’s highly educated Deaf community); and The Sinai Deaf Health program in Chicago, IL (selected because of its long-standing commitment to Deaf health education and research and its minority community).

### Recruitment

Recruitment occurs at each site through the use of flyers, email listserves, social media postings and vlogs (video-based blogs), and community outreach. Once a potential participant is deemed eligible, a research staff educates him or her on the study, and if interested in proceeding, an in-person research visit is scheduled. The informed consent is reviewed and signed at the in-person visit before proceeding with the study. The research staff who worked with the Deaf participants are fluent in ASL.

### Eligibility Criteria

To be eligible, study participants must be aged at least 18 years. For the Deaf study group, eligible Deaf participants must identify themselves as Deaf and communicate primarily in ASL. For the hearing comparison group, the hearing study participants also must be aged at least 18 years but self-report normal hearing and communicate in spoken English (ie, English monolinguals). Participants will be excluded if they have any cognitive impairments (eg, because of dementia, delirium, or intoxication), inability to consent to the study, or limited vision.

### Sample Size

To detect differences between correlations at 1% level of significance with 80% power, based on a one-sided Z-test of comparison of Fisher Z-transformed correlations, we plan to recruit 450 participants in each group that will allow us to detect a minimum difference of 20%. The sample size is more than adequate in detecting the differences between the groups with respect to the accessibility, acquisition, and attitude measures in aim 1 as well as to assess deafness as an effect modifier of the association between health literacy and ASK with health information.

### Measures

The constructs outlined in the Deaf Health Literacy conceptual model will be measured through a collection of chosen tools (see Table 1). These tools were selected primarily because of their accessibility (prior use with past projects involving Deaf participants) and their key constructs that they measure. There were several tools that required translation and adaptation before they could be used in the study. A translation work group (TWG) was formed for this purpose. ASL is a language with its own rules of semantics and syntax similar to other languages.

**Table 1:** Key variables and their measures.

Key variables		Measures	Defintion
**Attitudes**			
	Self-efficacy	One’s belief in one’s ability to succeed in specific situations.	Self-efficacy for information seeking [26].
**Skills**			
	Cognition	Ability to process information, metacognition, and behavioral regulation, which all are important for learning and social behavior	Kaufman Brief Intelligence Test, Second Edition (assesses nonverbal cognition), and the Color Trails Test (assesses executive function) [27] provide standard scores. Both have been used extensively in Deaf populations [28,29].
	Reading literacy	Level of reading proficiency	reading grade level from the Test of Silent Contextual Reading Fluency, Second Edition [30]. This instrument is visual and provides a suitable tool for both Deaf and hearing populations.
	Electronic health (eHealth) literacy	Ability to seek out, find, evaluate and appraise, integrate, and apply what is obtained in an electronic or Web-based environment to solving a health problem.	eHealth literacy Scale (validated measure of Web-based and eHealth literacy) [31]. Adapted further for the Deaf ASL^a^ users that includes whether Web-based information is accessible in ASL.
	Visualization of health information	Visualization patterns of the presented health information.	Actual through eye tracking software, which assesses the visualization of health-related information on 4 health topics [20,32].
**Knowledge**			
	Health knowledge	Knowledge of cardiovascular health.	Wagner et al’s cardiovascular health knowledge measure [33].
	Language fluency	Level of expressive and receptive language proficiency.	(1) ASL-SRT^b^ [34] and (2) Speaking Grammar Subtest of the Test of Adolescent and Adult Language, Third Edition [35]. Both will assess reception and expressive fluency in both ASL and spoken English. The ASL-SRT has been used extensively in Deaf populations [36,37].

^a^ASL: American Sign Language.

^b^ASL-SRT: American Sign Language Sentence Reproduction Test.

The shape, placement, and movement of hands as well as facial expressions and body movements all play important parts in conveying important information. There is no written form of ASL, so the use of *ASL gloss* is a way to keep note of how the ASL concepts should be signed to convey the meaning of the question or instructions accurately. Furthermore, the use of gloss has to do with the fact that the target language may not have equivalent words to represent the original language. The majority of the instruments were already available in ASL before the study’s funding, but there was translation and adaptation work needed for the following instruments: eHealth literacy Scale (eHEALS; 8 questions), Pew Research Center’s *Health Online Survey* questions related to online technology and information use (41 questions) [25], certain sociodemographic questions, cardiovascular knowledge assessment (25 questions), and self-efficacy instrument (3 questions).

The TWG consisted of the principal investigator (PI), a faculty experienced in translation of research instruments for Deaf individuals, 1 ASL interpreter skilled in medical terminology, and 3 native Deaf signers experienced with translation work. This translational workgroup approach was successful in the adaption of prior measures (eg, American Sign Language-Newest Vital Sign, ASL-NVS) [1]. All TWG team members are bilingually fluent in English and ASL, including expertise within their content area or translation work. Moreover, the team members have Deaf cultural issues as they are members of the Deaf community. The TWG members, including Deaf and hearing individuals, worked collaboratively in small groups to prepare, review, and perform quality checks. In addition to multiple intrateam reviews during the translation and filming process (videos used for internal reviews and back-translation steps), external reviewers, who were not members of the translation team, provided an added layer of review and validation to ensure the quality and integrity of the work. A final blind back-translation from the ASL videos back to the English text was done by 2 bilingual Deaf experts (not involved with the TWG) to ensure translation accuracy. The above steps, including selection of the team, were done to establish appropriate language and cultural translations.

#### Health Literacy Assessment

The study will use an accessible and validated health literacy instrument for Deaf individuals that the PI derived from the Newest Vital Sign (referred to as the ASL-NVS) [38]. The ASL-NVS also offers other benefits as it assesses numeracy and reading literacy, has a short administration time with only 6 questions, and has been created and validated for other languages (eg, Spanish). The ASL-NVS is the first, and currently only, health literacy instrument available for the Deaf population. The instrument is available online [39].

#### Attitudes Assessment

##### Self-Efficacy

The *Self-efficacy for Information Seeking* scale is based on a scaled 3-item questionnaire assessing beliefs about ability to effectively perform specific information-related behaviors, such as finding information about health, evaluating the accuracy of health information, and using the internet to find information [26].

#### Skills Assessments

##### Nonverbal Cognitive Ability

The *Kaufman Brief Intelligence Test, Second Edition* (KBIT2) [40], Matrices Subtest (nonverbal intelligence quotient [IQ]) is computed based on one’s performance on a single multiple-choice subtest that does not require language skills. There are 46 items composed of several types of items involving visual stimuli, both meaningful (people and objects) and abstract (designs and symbols). All items require understanding of relationships among the stimuli and require the participant to point to the correct response. The KBIT2 nonverbal IQ has a 0.82 correlation with the Wechsler Adult Intelligence Scale, Third Edition, nonverbal composite [40]. This test has recently become commonly used in cognitive science and psycholinguistic studies that have included Deaf participants [21,22,29] to control for individual cognitive differences.

##### Executive Function Ability

The *Color Trails Test* (CTT) [27] is a measure of executive function abilities where participants are asked to rapidly connect numbered circles in sequence by alternating between pink and yellow circles [27]. The time to complete the task is recorded. The CTT has a test-retest reliability of 0.79 and has good factorial and criterion-related validity [27] with individuals with frontal lobe injuries. One of the coinvestigators (PCH) has used this test in cognitive science studies involving Deaf participants [28,29].

##### Reading Literacy

This will be assessed through the Test of Silent Contextual Reading Fluency, Second Edition [30]. This tool provides an efficient standardized literacy assessment for both Deaf and hearing participants.

##### Electronic Health Literacy

*eHEALS* was adapted to make it more culturally and linguistically appropriate for Deaf ASL users. The 8-item questionnaire inquires about the participants’ knowledge, comfort, and perceived skills at finding, evaluating, and applying eHealth information to health problems [31]. An additional 41 questions derived from the Pew Research Center’s *Health Online Survey* [25] related to online technology and information will assess the participant’s level of information access and use. This also was expanded to inquire about ownership of computer and mobile-based devices.

##### Health Information Evaluation Skill

Actual behaviors will be assessed for each participant through the use of the eye tracking software (Tobii; Tobii Technology) on 4 standardized health topic pages (2 with a picture and 2 without a picture). Eye tracking technology will be used to observe their ability to visualize and understand standardized health information with and without pictures. For eye tracking, we will use the Tobii eye tracker and software for data collection on fixation duration and fixation count on the presented information and the visualization pattern on both websites. This eye tracker provides extremely detailed eye tracking metrics (in milliseconds), including fixation counts, visit duration, time to first fixation, and percentage of viewing time fixated/clicked on a certain area of interest. It also can be used in either static (eg, print) or dynamic (eg, video or website scrolling) to assess each participant’s areas of interest.

#### Knowledge Assessments

##### Health Knowledge

A validated, general health knowledge test called the Heart Disease Fact Questionnaire [33] will be used. It assesses knowledge of cardiovascular health in a true/false/do not know questionnaire with 25 questions. There is a paucity of general health knowledge assessments that are validated. This tool is one of the few that provides a range of topics to assess participants’ knowledge and has been used in a previous study involving Deaf individuals.

##### Language Fluency

As Deaf individuals reside in communities that use English as the *lingua franca* (or *de facto* language), they are frequently bilingual. Fluency will be assessed in both ASL and English for the Deaf participants. English will be assessed for the hearing participants. ASL fluency will be tested with the *American Sign Language Sentence Reproduction Test* (ASL-SRT) [34], which is a brief global measure of individuals’ receptive and expressive ASL skills. Participants are required to watch videos of 20 ASL sentences and correctly reproduce the sentences after each one is presented. Correct reproductions are awarded 1 point, and the maximum total score is 20. ASL-SRT has been used in other cognitive science and psycholinguistic studies [36,37] and has been adapted to other signed languages. The ASL-SRT interrater reliability coefficient is 0.83, with an internal consistency (Cronbach alpha) of .88. CConstruct validity was established by illustrating that Deaf adults perform better on this test than Deaf children (*P*<.001; partial eta sq=.042) and native signers perform better than nonnative signers (*P*<.001; partial eta sq= 274). An equivalent and parallel test is available that assesses the receptive and expressive spoken English abilities that will be used for hearing participants. The test is called *Speaking Grammar Subtest of the Test of Adolescent and Adult Language, Third Edition* (TOEL3) [35], and has been widely used for language fluency assessments. The TOEL3 has internal consistency (Cronbach alpha) of .95, with test-retest reliability coefficient of 0.80.

Sociodemographic information will be based on an existing shortened version of the Behavioral Risk Factor Surveillance Systems [41]. This will be used to evaluate key demographic information for each participant and adjust for covariates in health literacy. For Deaf participants, additional questions will be asked to provide information on the following: (1) presence of Deaf family members, (2) ownership of a hearing aid or cochlear implant, (3) hearing status with and without a hearing aid or cochlear implant, (4) onset of hearing status, (5) any cultural identity with their hearing status, (6) Deaf school attendance, and (7) socialization preferences based on hearing status (Deaf, hearing, or both). We will also perform a Shoebox Audiometry [42] on all participants to assess their unaided hearing levels (HLs) from (−)10 dB to 90 dB at 500 Hz, 1000 Hz, 2000 Hz, and 4000 Hz for each ear. Shoebox is a leading tablet-based audiometer, which is a class 2 medical device registered and listed with the Food and Drug Administration. It offers a clinically validated, self-administered, automated testing platform optimized for use outside of a sound booth; can be completed in 5 min or less; and is completely configurable. HL will be defined by the standard greater than or equal to 25 dB average loss across the 4 frequencies in the better ear [43]. This will allow us to control for hearing loss variations among participants, especially because they may influence the level of access to information.

### Procedures

Once the participants are eligible, the research team will schedule an in-person visit to review both the informed consent and administer the different measures. This was done in the same fashion at each performance site. The research staff review the consent form and the brief study information directly in the participant’s preferred language (eg, ASL if Deaf). Once consent is obtained, the staff then proceeds with the data collection protocol. The measures are grouped into the following categories: (1) demographics/background information (includes Shoebox Audiometry), (2) interviewer-administered questionnaires (ie, cardiovascular knowledge assessment, self-efficacy, Web-based health information use, and e-literacy), (3) computer-administered measures (ASL-SRT, TOEL-3, and the ASL-NVS), (4) booklet testing (ie, CTT, KBIT2, and TOSCRF-2), and (5) Tobii eye tracker experiment. To avoid any ordering bias, the categories (except for the demographic/background information) are randomized to each participant (see Multimedia Appendix 1). The computer-generated randomization was completed before any participant recruitment. The counterbalance checklist designed with 4 blocks (A, B, C, and D) was randomly assigned to each participant (eg, 1 participant may get a checklist C that starts with booklet testing).

#### Procedures for Return Visit (for Those to Be Invited)

There will be 2 parts when the participant returns for their second visit (see Table 2). The first step will observe how participants search and acquire Web-based health information. This task performance will include 4 brief clinical vignettes (ie, pneumonia, deep vein thrombosis, migraines, and appendicitis) and multiple-choice answers will serve as a prompt. This will be given to all invited aim 3 participants to examine their abilities to search for health information on the Web and acquire health knowledge. Participants’ query formulations, navigation patterns, cursor activity, total search times, and number and nature of websites accessed to address the clinical vignettes will be recorded and assessed through the use of the Tobii usability testing software already available in our facilities [44]. The data will include video footage of the participant (including eye movements and facial expression), websites visited, duration of page visited, and number of clicks and cursor activity. The use of the Tobii provides several benefits: (1) the tool provides real-time dual-screen recording and display of both the participant’s actions online and webcam recordings of their facial expressions and eye movements; (2) the interviewer can watch the actions of the participant on the interviewer’s computer screen through the use of the Tobii Pro Studio software program in real time; (3) the interviewer can tag different-colored annotations (eg, search queries, websites accessed, and challenges encountered) to the video data being recorded, which is visible on the time bar; (4) the tool can instantaneously compute summary statistics such as the total search time in seconds, the number of unique websites and pages visited, and the number and characteristics of queries issued for the interviewer to discuss with the participant; and (5) the interviewer can scroll quickly to the relevant time of the video to help the participant observe and remember their actions but, more importantly, allow for the interviewer to ask more detailed questions on how and why specific actions were chosen and the participant’s thought process related to the online information. A figure demonstrating the different components and how it works can be accessed at Tobii website.

The recordings from the task performance above will serve as a prompt for the elicitation interview. It will help inform the subsequent qualitative assessment to help explain the quantitative results (of aims 1 and 2) and elucidate how and why Deaf individuals access and understand health information. Similarly, the same approach will be conducted with a subsample of hearing individuals. This will be done to provide robust comparisons. Video-based and semistructured qualitative assessments will be used to determine both how and why Deaf and hearing participants of different health literacy strata (inadequate and adequate; n=15 each) may differ in their performances with Web-based health information searches. The findings here will be used to help explain results from aims 1 and 2. The elicitation interview questions will be generated initially from the findings in aims 1 and 2 and complemented with additional questions following completion of the task performance on a computer. The interviewer will playback the dual-screen recording of the participant’s actions to facilitate recall of his or her own actions and thoughts with each task undertaken. The interviewer will encourage the participant to make his/her thoughts transparent, that is, telling how and why the choices were made in a narrative format. The dual-screen recording with performance metrics will serve as stimulus material for the video elicitation interview. As the dialog between the interviewer and the participant will be in ASL (visual language), a separate external video camera will be used to record this interaction for later transcription. The interview will reveal the complex cognitive and decision-making processes that may occur with each individual, which are not adequately revealed in earlier statistical analysis. The incorporation of both steps provides better integration of the findings of all 3 aims and generates robust data to guide future intervention development to address inadequate health literacy and information marginalization for Deaf populations. A strength of this step is the ability to explore contextual factors (ie, task- and user-oriented factors) that could not be explained with the first visit. As stated by a newer eHealth literacy model [45], these task- and user-oriented factors are important in their roles in the overall eHealth context. The proposed step will help examine how these intrapersonal and system-based factors may shape the Web-based health experience and abilities for both Deaf and hearing individuals.

**Table 2:** Aim 3 approaches.

Definition	Purpose	Approach	Data collection	Expected outcomes
Web-based health information navigation and comprehension (step 1)	To assess Web-based health information search ability, acquisition, and patterns	Use of Tobii video/online use recording software; 4 clinical vignettes with questions to prompt Web-based search	Navigation patterns of Deaf individuals; preferences of health websites, including search engines (n=15 Deaf and n=15 hearing for comparison); scoring on questions related to vignettes	Assess Web-based health information search ability of Deaf individuals (unknown); determine if Deaf individuals’ health literacy determines the quality of the navigation and comprehension of the information (unknown); generate areas of weaknesses and strengths for interventions to focus (unknown)
Elicitation interviews (step 2)	To explain and expand the understanding of the findings demonstrated in aims 1 and 2	Use of elicitation interviews about video recordings of the participants and their Web-based searches	Explore how and why participants decided to use the websites they chose, including the types of queries and what aspects of the Web-based information was useful to them	Assess how Deaf individuals visualize and learn Web-based health information (unknown); determine the preferences of health information tools and dissemination (unknown) and understand why they have those preferences (unknown)

### Statistical Analyses for Aim 1

Frequency statistics will be used to summarize categorical data, whereas means (SDs) were computed to describe continuous data. Correlations between main study variables will also be analyzed using Pearson r correlation coefficients. Differences between the Deaf and hearing participants in terms of ASK related to health information will be assessed using linear regression models. To control for the effects of demographic and physiological covariates on the dependent variables of ASK related to health information, sociodemographic (eg, race) and physiological factors (ie, hearing loss severity and laterality) will be entered in model 1 as potential confounding factors affecting each variable of interest. As we are primarily interested in the additional effects of ASK related to health information on health literacy and eHealth, predictor variables will be subsequently entered in model 2. Differences between the Deaf and hearing participants in terms of ASK related to health information will be assessed using linear regression models. The scales for each modality described in Table 1 will be used as a separate outcome with Deaf versus hearing as the primary covariate. A propensity-adjusted analysis will also be conducted with the propensities defined as the predicted probability of having adequate health literacy as a function of sociodemographic factors estimated through a logistic regression model. For observational data, the propensity-matched methods are often deemed to be a better alternative to the usual multiple linear regression controlling for potential confounders. Strata based on propensity quantiles will be used as a categorical covariate in the linear regression model. Furthermore, the variability across sites will be controlled for in the model. Usual model diagnostics will be performed, followed by corrective actions as necessary.

### Statistical Analyses for Aim 2

The ASL-NVS and the English-NVS will be used to assess the health literacy score in both Deaf and hearing participants, respectively. The health literacy indicator (ASL-NVS) ranges in value from 0 to 6. To understand whether the association between health literacy and ASK related to health information differs between Deaf and hearing individuals, we shall compute in the Deaf and hearing groups Fisher Z-transformed partial correlations between health literacy and each of the constructs, adjusted for the socioeconomic factors and potential site differences. Subsequently, 2-sample Z-test of these correlations will be carried out for each construct. We shall supplement this analysis with a regression approach where health literacy will be regressed on the group and each of the constructs along with their interaction controlling for the socioeconomic factors and site. Although the interaction term is of primary focus, such a model will provide an overall appraisal of the association between health literacy and various covariates. On the basis of PI’s previous work involving the ASL-NVS, one can alternatively characterize health literacy as a categorical variable with categories of adequate (NVS score of 5-6), indeterminate (2-4), and inadequate (0-1). As a secondary analysis, we will conduct an ordinal logistic regression with the categorical outcome of health literacy using the same covariates in the model as mentioned above.

### Analyses for Aim 3

Building on the methodology used in previous Web-based search behavior studies [46], participants’ search skills and success in addressing the clinical scenario tasks will be assessed using a combination of methods. First, based on the browser log and interaction data gathered by the usability software, we will compute summary statistics, such as the total time (in minutes) spent in searching, the number of unique websites and pages visited, and the number and characteristics of queries issued, involved in completing each task. Second, we will include time-based retrieval measures [47] that can characterize and quantify how a participant’s search progress evolves over time. Knowledge acquisition will be measured by the number of correct responses based on the clinical vignettes (score range between 0 and 12). All measures representing navigation skill and knowledge acquisition will be compared across levels of health literacy (adequate vs intermediate/inadequate) on the subgroup of the preselected 30 Deaf participants using 2 sample *t* tests. Although it is anticipated that power will be low to observe statistical significance, the primary objective for this comparison is to identify trends. Search progress for the clinical scenarios will be defined in terms of the quality of health information pages found during the search task. To assess the quality of health information pages, the research team will evaluate the Web pages/resources returned by participant searches or visited by the participant via browser navigation in terms of accuracy and coverage of the target facts in the reference set. Any differences will be discussed until a consensus is obtained.

### Analysis of the Elicitation Interviews

For the iterative analysis of the qualitative data obtained with the elicitation interview process, we will follow multiple steps: (1) thoroughly read the transcribed data, (2) generate initial codes, (3) search for themes and patterns among the codes and across participants, (4) review themes, and (5) define and name themes. Initial data queries will focus on questions that emerge from aims 1 and 2 and navigability and knowledge acquisition patterns as measured in step 1. As we learn more through the iterative elicitation interview/analysis process, emerging themes and questions will be explored. Video-recording data of the one-on-one interviews will be transcribed into English by a bilingual transcriber [10]. Field notes will be taken after each encounter to describe the context, primary content, and emerging concepts. Participants’ data in aim 3 will also be linked with the data collected in aims 1 and 2 that helped inform questions in the elicitation interviews. This will be done through the use of joint displays, a state-of-the-art integration procedure in mixed methods studies used for visually matching results by domains and themes from the quantitative and qualitative data to link the relevant information. All raw data video files will be uploaded onto a secure server, transcribed at the PI’s office, and analyzed by the qualitative team. The use of Dedoose software [48] will allow transcription and detailed annotation to be tagged with video data of Deaf signers. All qualitative data (transcripts and field notes) will be coded independently by multiple investigators. Codes will be developed using consensus. A codebook will be created initially using navigation and knowledge acquisition and will be expanded with emergent codes. All transcripts will be coded by the research team (shown above) using the codebook in Dedoose.

## Results

The project received human participants approval from the University of Michigan IRB (HUM00132918) to provide oversight to the 3 other research sites. Data collection will be completed in late 2020. Findings from this study will be used to advance what is known about health literacy and health information accessibility for the Deaf population. The findings will help explain how Deaf individuals access, navigate, and comprehend Web-based information. This study will help guide formulation of health information and health literacy interventions that more accurately take advantage of visual learning skills, even for those who have normal hearing. The findings will also be potentially beneficial for website editors. On the basis of the findings, recommendations for improving accessibility to health information for those who are non-English speaking and for those with inadequate health literacy will be provided. Results will be published in peer-reviewed journals once completed.

## Discussion

### Principal Findings

This project will advance knowledge on the origins of inadequate health literacy among Deaf individuals, including how effectively they access Web-based health information. In this paper, methods developed for the study will help assess the role of information marginalization on health literacy in the Deaf population. We also describe our approach to adapt instruments and measures applicable for Deaf ASL users. This project is the first to systematically explore health literacy for Deaf individuals. Most Deaf studies focus on Deaf health knowledge gaps and disparities, but our goal is to provide tangible recommendations going forward, especially for those involved with health information dissemination and website editors.

### Direct Impact

Improvement in translation work and their processes can have an important impact as it affects other funded research projects and those that are being proposed. Furthermore, existing measures (eg, ASL-NVS) will be shared with other investigators interested in working with this population. We hope that the measures can be incorporated into community and clinical surveillance to help them manage their own health and determine how health information may or may not be reaching these individuals. Research with these communities are highly underfunded. This project provides an example of feasible research with the Deaf community. In addition, little is known about the predictors of health literacy in Deaf populations and how this affects their ability to use Web-based health information. This is key because of their social and information marginalization and their documented risk for inadequate health literacy. Web-based health information, if accessible, can provide a strategic approach to address the community’s health knowledge gaps.

The results of this mixed methods proposal will significantly advance what is known about health literacy and health information accessibility for the Deaf population. This innovative study will generate rich data on how to formulate health information and health literacy interventions more accurately to take advantage of visual learning skills. The study results will be provided once data collection is complete (anticipated in 2020). Once finalized, findings will be used to develop a white paper on how to structure Web-based health information in a way that maximizes accessibility and comprehension for Deaf individuals and also includes strategies that adhere to universal health literacy precautions because of the Deaf population’s documented risk of inadequate health literacy.

### Timeline and Challenges

It is important to establish adequate time and resources to ensure adequate translation and adaptation of instruments needed in the project. This process took longer than anticipated. It also requires a diverse team to ensure good comprehension. We also needed to do 1 additional step and elicit feedback from the advisory board and the staff member at each site to make sure there were no issues (eg, regional variations for certain signs). It was decided that interviewer-administered questionnaires were preferred because of participant’s different levels of language fluency. Although this did not yield a computerized self-administered tool, it was done to reduce any potential data collection errors or language/cultural discordance with Deaf participants. In addition to the TWG, our research team required extensive training to ensure strict protocol adherence, given the multiple study measures. In preparation for going into the field, we conducted both a site-specific workshop and an in-person workshop with all staff and investigators together to standardize interviews, administration of instruments, and data entry required for the study. Furthermore, to avoid participant fatigue with multiple measures, we plan to offer refreshments and ample opportunity for breaks during the data collection visits.

### Conclusions

Research involving Deaf individuals requires careful consideration of instruments and measures that will not cause measurement bias, cultural discordance, and inaccessibility issues. Furthermore, dedicated time for adaptation, translation, and, if needed, validation steps should be factored when calculating the appropriate research time and funds needed to implement the project. Adequate training time is needed when conducting a multisite study involving several research staff. The results from this mixed methods proposal will advance what is known about health literacy and health information accessibility for the Deaf population. This study will also be useful to develop best practices in improving the accessibility and usefulness of Web-based health for individuals at risk for inadequate health literacy.

## Appendix

Multimedia Appendix 1 Random ordering of measures.

Multimedia Appendix 2 Peer-reviewer report from the National Institutes of Health.

## Abbreviations

ASK: attitudes, skills, and knowledge
ASL: American Sign Language
ASL-NVS: American Sign Language-Newest Vital Sign
ASL-SRT: American Sign Language Sentence Reproduction Test
CAB: community advisory board
CTT: Color Trails Test
eHEALS: eHealth literacy Scale
eHealth: electronic health
HL: hearing level
HMC: Hurley Medical Center
IQ: intelligence quotient
IRB: institutional review board
KBIT2: Kauffman Brief Intelligence Test, Second Edition
NTID: National Technical Institute of the Deaf
NVS: Newest Vital Sign
PI: principal investigator
TOEL3: Speaking Grammar Subtest of the Test of Adolescent and Adult Language, Third Edition
TWG: translation work group
