# Supplementary material for: Deaf Adults’ Health Literacy and Access to Health Information: Protocol for a Multicenter Mixed Methods Study
Source: JMIR Res Protoc. 2019 Oct 9;8(10):e14889. doi: 10.2196/14889 (PMC6812478; doi:10.2196/14889)
Supplement: Multimedia Appendix 1 [file resprot_v8i10e14889_app1.docx]

**Checklist A**

| - Informed consent, - Ask where they learned about the study |
| --- |

| Background |
| --- |
| - Sociodemographic questionnaire - Shoebox Audiometry |

| Computer measures |
| --- |
| - ASL-SRT/TOEL-3 (Language Flueny) - NVS (Health Literacy) |
|  |
|  |

| Booklet testing |
| --- |
| - TOSCRF-2 (Reading literacy) - CTT (executive function assessment) - KBIT (non-verbal IQ) |
|  |
|  |

| Eye-tracking measures |
| --- |
| - Tobii A   - Set up webcam to record   - Monitor/seat check   - Medical Topic Survey (assess their familiarity with the topic)   - Flu practice (helps participant to get acquainted with the Tobii)   - Conduct Testing |
|  |

| Questionnaires |
| --- |
| - Cardiovascular knowledge assessment (assesses their knowledge level) - Self-efficacy - Online health information use and e-literacy |

| Closing |
| --- |
| - Provide incentive and sign receipt |

**Checklist B**

| - Informed consent - Ask where they learned about the study |
| --- |

| Background |
| --- |
| - Sociodemographic questionnaire - Shoebox Audiometry |

| Eye-tracking measures |
| --- |
| - Tobii B   - Set up webcam to record   - Monitor/seat check   - Medical Topic Survey (assess their familiarity with the topic)   - Flu practice (helps participant to get acquainted with the Tobii)   - Conduct Testing |
|  |

| Computer measures |
| --- |
| - ASL-SRT/TOEL-3 (Language Flueny) - NVS (Health Literacy) |
|  |
|  |

| Booklet testing |
| --- |
| - TOSCRF-2 (Reading literacy) - CTT (executive function assessment) - KBIT (non-verbal IQ) |
|  |
|  |

| Questionnaires |
| --- |
| - Cardiovascular knowledge assessment (assesses their knowledge level) - Self-efficacy - Online health information use and e-literacy |

| Closing |
| --- |
| - Provide incentive and sign receipt |

**Checklist C**

| - Informed consent - Ask where they learned about the study |
| --- |

| Background |
| --- |
| - Sociodemographic questionnaire - Shoebox Audiometry |

| Booklet testing |
| --- |
| - TOSCRF-2 (Reading literacy) - CTT (executive function assessment) - KBIT (non-verbal IQ) |
|  |
|  |

| Eye-tracking measures |
| --- |
| - Tobii C   - Set up webcam to record   - Monitor/seat check   - Medical Topic Survey (assess their familiarity with the topic)   - Flu practice (helps participant to get acquainted with the Tobii)   - Conduct Testing |
|  |

| Computer measures |
| --- |
| - ASL-SRT/TOEL-3 (Language Flueny) - NVS (Health Literacy) |
|  |
|  |

| Questionnaires |
| --- |
| - Cardiovascular knowledge assessment (assesses their knowledge level) - Self-efficacy - Online health information use and e-literacy |

| Closing |
| --- |
| - Provide incentive and sign receipt |

**Checklist D**

| - Informed consent - Ask where they learned about the study |
| --- |

| Background |
| --- |
| - Sociodemographic questionnaire - Shoebox Audiometry |

| Computer measures |
| --- |
| - ASL-SRT/TOEL-3 (Language Flueny) - NVS (Health Literacy) |
|  |
|  |

| Eye-tracking measures |
| --- |
| - Tobii D   - Set up webcam to record   - Monitor/seat check   - Medical Topic Survey (assess their familiarity with the topic)   - Flu practice (helps participant to get acquainted with the Tobii)   - Conduct Testing |
|  |

| Booklet testing |
| --- |
| - TOSCRF-2 (Reading literacy) - CTT (executive function assessment) - KBIT (non-verbal IQ) |
|  |
|  |

| Questionnaires |
| --- |
| - Cardiovascular knowledge assessment (assesses their knowledge level) - Self-efficacy - Online health information use and e-literacy |

| Closing |
| --- |
| - Provide incentive and sign receipt |
