# Supplementary material for: Deaf Adults’ Health Literacy and Access to Health Information: Protocol for a Multicenter Mixed Methods Study
Source: JMIR Res Protoc. 2019 Oct 9;8(10):e14889. doi: 10.2196/14889 (PMC6812478; doi:10.2196/14889)
Supplement: Multimedia Appendix 2 [file resprot_v8i10e14889_app2.pdf]

SUMMARY STATEMENT
(Privileged Communication)

PROGRAM CONTACT:
Amy Donahue
(301) 402-3458
amy_donahue@nih.gov

Release Date: **05/14/2015**
Revised Date: **06/02/2015**

Application Number: **1 R01 DC014703-01A1**

Principal Investigator

MCKEE, MICHAEL M MD

Applicant Organization: UNIVERSITY OF MICHIGAN

Review Group: **ZRG1 HDM-X (56)**
Center for Scientific Review Special Emphasis Panel
PAR13-132: Understanding and Promoting Health Literacy

Meeting Date: **05/08/2015**
Council: **OCT 2015**
Requested Start: **09/01/2015**

RFA/PA: **PAR13-130**
PCC: **HR05**
Dual IC(s): **MD**

Project Title: **Mechanisms of Health Literacy and Information Accessibility in the Deaf**

SRG Action: **Impact Score: 20 Percentile: 5**
Next Steps: **Visit http://grants.nih.gov/grants/next_steps.htm**
Human Subjects: 30-Human subjects involved - Certified, no SRG concerns
Animal Subjects: 10-No live vertebrate animals involved for competing appl.
Gender: **1A-Both genders, scientifically acceptable**
Minority: **1A-Minorities and non-minorities, scientifically acceptable**
Children: **1A-Both Children and Adults, scientifically acceptable**
Clinical Research - not NIH-defined Phase III Trial

Project Year	Direct Costs Requested	Estimated Total Cost
1	457,798	679,194
2	438,390	650,400
3	438,290	650,251
4	474,147	703,449
5	440,648	653,750
TOTAL	2,249,273	3,337,043

ADMINISTRATIVE BUDGET NOTE: The budget shown is the requested budget and has not been adjusted to reflect any recommendations made by reviewers. If an award is planned, the costs will be calculated by Institute grants management staff based on the recommendations outlined below in the COMMITTEE BUDGET RECOMMENDATIONS section.

EARLY STAGE INVESTIGATOR, NEW INVESTIGATOR

EARLY STAGE INVESTIGATOR

NEW INVESTIGATOR

COMMITTEE BUDGET RECOMMENDATIONS

RESUME AND SUMMARY OF DISCUSSION: This application requests support to conduct a multi-site research project to study attitudes, knowledge and skills, as well as other factors, such as cognitive abilities, resilience and self-efficiency that may lead to low health literacy among American Sign Language (ASL) user populations. If successful, the findings will potentially inform and guide how health information is crafted and delivered to deaf individuals with the potential for reducing the existing health disparities experienced by this vulnerable group. All of the weaknesses identified in the previous review were well addressed by providing a leadership and study management plan, adjusting roles and effort of the team and providing further detail on the approach: the study now has a high potential impact. In discussion, reviewers identified application strengths that include a potentially high impact, theory-driven design with high innovation, including a “measures translational workgroup” and, an eye-tracking component; high potential for practical impact on health communication targeted to deaf and other visual learners; as well as, a diverse team with demonstrated sensitivity and knowledge of deaf culture. Reviewers also identified minor weaknesses. These include concerns regarding survey burden; potential for differential drop out between deaf and hard-of-hearing participants; use of self-report measures; and, potential for the hawthorn effect. Reviewers also noted that provision of a justification for selection of clinical scenarios would strengthen the application. Overall, this is an outstanding application with a high potential impact on health literacy of an underserved, vulnerable population of ASL users that far outweighs the noted minor, addressable weaknesses.

DESCRIPTION (provided by applicant): Deaf American Sign Language (ASL) users are nearly seven times more likely to have inadequate health literacy when compared with their hearing peers. This population is the non-English speaking minority group at greatest risk for miscommunication in health care settings. Health literacy mechanisms for Deaf individuals remain poorly understood, thereby limiting interventions to address health literacy disparities and their impact on health care. It is unclear how differences in attitudes, knowledge, and skills related to health information affect health literacy in Deaf populations and how they may contribute to ongoing health inequities. The two primary objectives of this proposal are: 1) to elucidate the role of information marginalization on health literacy in Deaf American Sign Language (ASL) users and 2) to better understand the mechanisms of health literacy in this population so as to identify viable targets for future health literacy intervention development. This proposal is responsive to PAR-10-133's request for studies that assess mechanisms underlying health literacy, including roles of cognition, culture, language fluency, and information-seeking and interpretation ability in the deaf population and, how these may differ from the hearing population. To meet the study objectives, we will employ an explanatory sequential mixed methods design using extensive quantitative data collection procedures, namely, cross-sectional surveys and measures that will identify predictors and moderators of health literacy with 450 Deaf and 450 hearing subjects across three geographically diverse sites. These results will inform the subsequent qualitative assessment that will help explain the quantitative results, and elucidate how and why Deaf individuals access and understand health information. We will incorporate cutting edge technology to assess health information-seeking and interpretation patterns in this population, in addition to using a variety of validated and ASL -accessible instruments to assess health literacy and other constructs related to health literacy. The diverse team, consisting of both leading deaf and hearing researchers, provides a unique insight into how health information is distributed and disseminated visually. This approach has the potential to generate rich data on how to formulate health information and health literacy interventions for individuals with hearing loss.

PUBLIC HEALTH RELEVANCE: The public health impact of this study is its ability to understand potential differences in attitudes, knowledge, and skills related to health information between deaf and hearing populations and their degree of impact on health literacy. This research will assist us with improving existing health literacy interventions and tailor future approaches suitable to address the ongoing health inequities seen in the deaf population.

Critique 1

Significance: 1
Investigator(s): 2
Innovation: 1
Approach: 3
Environment: 1

Overall Impact: The study examines information marginalization on health literacy in Deaf American Sign Language (ASL) users and aims to better understand the mechanisms of health literacy in this population for the purpose of identifying viable targets for future health literacy intervention development. Study findings will significantly advance understanding of the mechanisms underlying health literacy and determine the causal pathways that link health literacy to behavioral outcomes for Deaf individuals. The proposal places a particular focus on the following areas relevant to Deaf individuals: analyzes and measures the abilities and skills required to be functionally health literate; elucidates the barriers and factors involved in the comprehension of health information; evaluates the effect of information technologies on health literacy; examines the role of linguistic ability and skills, along with cultural factors in non-native English speakers; and examines the moderators of health literacy on information-seeking and interpretation abilities. The study is significant, innovative, and employs reliable methodology.

1. Significance:

Strengths

- The proposed study is conducted in a highly vulnerable population of Deaf patients who due to their extreme social and language marginalization and variety of communication barriers resort to online health information more frequently than the general population
- This mixed methods proposal will significantly advance what is known about health literacy and health information accessibility for the Deaf by integrating the roles of attitudes, skills, and knowledge related to health information with video-elicitation interviews of how Deaf individuals' access, navigate and comprehend online information.
- Since Deaf individuals communicate through a visual language, this study is significant in that is provides an opportunity to determine optimal visual-based information sources.
- The investigators have conducted significant amount of work demonstrating higher rates of inadequate health literacy among Deaf individuals compared to hearing individuals
- Study may also provide novel information for the general English speaking population who are visual learners and visually based online health information.

Weaknesses

- NA

2. Investigator(s):

Strengths

- The principal investigator has displayed strong academic track record as demonstrated by his publications and his research implementation
- The assembled team provides expertise in health literacy research and causal pathways development, expertise in language acquisition and cognitive development, statistical and data management and oversight, mixed methods
- The assembled research team, three Deaf researchers and a Deaf community advisory board, provides expertise to be able to measure a variety of constructs in a culturally and linguistically appropriate manner.
- The PI and multiple co-investigators have strong collaborations with a variety of Deaf-based organizations, including the National Center for Deaf Health Research and its community-based partner, Partners in Deaf.

Weaknesses

- A supportive advisory board may help with the perspective

3. Innovation:

Strengths

- This is an innovative study that will generate rich data on how to formulate health information and health literacy interventions more accurately to take advantage of visual learning skills.
- Includes the focus on potentially important determinants, including the role of cognition, language, resilience, and accessibility of health information in a vulnerable and marginalized population
- Defines and comprehensively examines a healthy literacy mechanism for the Deaf, which has the potential to become a landmark framework for future work (socio-demographics, cognition, educational achievement including reading skills and task performances, and general health knowledge and how they impact accessing health information and building health literacy are not known among the Deaf)
- The proposal adequately incorporates new technologies including eye gaze and online search tracking that will improve understanding of how health information may be a moderator of health literacy for deaf individuals

Weaknesses

- None noted.

4. Approach:

Strengths

- Utilizes explanatory sequential mixed methods design using extensive quantitative data collection procedures such as cross-sectional surveys and measures that will identify predictors and moderators of health literacy with Deaf and hearing subjects.
- Assures that the survey instruments and measures used in the study will be adequately translated
- Uses valid and reliable instruments and methodology to measure heath literacy and health information accessibility (e.g., eye-tracking software, dual screen recording, the number of unique web sites and pages visited, and the number and characteristics of queries, elicitation interviews)

- Utilizes big sample size of 450 Deaf individuals, ensuring that study results will be generalizable

Weaknesses

- Potential survey burden to patients but investigators defend the need and time for administering different questionnaires
- Not clear whether investigators comment on the potential for differential participation by deaf and by hearing participants (deaf more likely to stay in study; how will hearing participants be engaged to stay in study)
- Various use of technology may be a great way to assess causal relationships but its use may be conducive to creating too much of a control environment in which patients may be more likely to find and access health information, knowing that they are monitored. Not clear whether investigators think that study results will be generalizable to real-world behavior of deaf patients
- When administering the four clinical vignettes, participants' prior knowledge of the disease or having a disease diagnosis related to the clinical vignette's disease area may impact the extent to which they will search, find, and access disease-specific information.

5. Environment:

Strengths

- Strong environment and diverse resources available to investigators to successfully undertake and complete the proposed work.

Weaknesses

- None noted.

Protections for Human Subjects:

Acceptable Risks and/or Adequate Protections

Data and Safety Monitoring Plan (Applicable for Clinical Trials Only):

Not Applicable (No Clinical Trials)

Inclusion of Women, Minorities and Children:

- Sex/Gender: Distribution justified scientifically
- Race/Ethnicity: Distribution justified scientifically
- Inclusion/Exclusion of Children under 21: Excluding ages < 21 justified scientifically

Vertebrate Animals:

Not Applicable (No Vertebrate Animals)

Biohazards:

Not Applicable (No Biohazards)

Revision:

- Investigators successfully address reviewer's comments from the previous submission

Budget and Period of Support:

Recommend as Requested

CRITIQUE 2

Significance: 1
Investigator(s): 1
Innovation: 2
Approach: 2
Environment: 1

Overall Impact: This revised R01 application from an Early Stage Investigator from the University of Michigan proposes to study health literacy among deaf adults and older adolescents. The well designed research proposal addresses a significant gap in the literature. The mixed methods approach is innovative and the approach is well described and planned. The investigative team is comprehensive and the senior investigators in the team will help guide the less experienced PI in a research environment that is complementary and supportive. A particular strength is the PIs involvement in the deaf community and the choice of collaborating institutions. While this is otherwise an exceptional proposal, enthusiasm is diminished by the failure of the application to address possible misclassification from the use of self-reports on hearing, the need for a stronger justification for the clinical scenarios, and the lack of a dissemination plan even though the investigators plan a community advisory group. In spite of the limitations, the proposed research has the potential, if well executed, to have a large and significant impact on the field.

1. Significance:

Strengths

- The application addresses an important public health problem and makes a strong and convincing case of the role of health literacy. The proposal is responsive to the program announcement.

Weaknesses

- The failure of the investigator to address the validity of self-reported hearing detracts from an otherwise thorough review and statement of the problem.

2. Investigator(s):

Strengths

- The PI is a well-trained and productive Early Stage Investigator whose K research is directly relevant to this application who completed his training in family medicine and preventive cardiology and public health in 2010. He was appointed as Assistant Professor in Family Medicine at the University of Michigan in 2013. He lists 10 first author publications and documents preliminary studies directly related to the proposed research. He is deaf and has developed strong relationships with the collaborating institutions and investigators that strengthen this application. The personal commitment of the PI to the deaf community is impressive.
- The limited experience of the PI is offset by increases in effort among co-investigators with substantial records of achievement.

- The revised application provides a plan for how the investigators and sites will work together and how this project can be effectively managed.

Weaknesses

- None noted.

3. Innovation:

Strengths

- The focus on health literacy in the deaf addresses a major gap in existing knowledge and practice. If successful, the research will have great public health and clinical utility.
- The use of mixed methods is appropriate.

Weaknesses

- The proposal would have been more innovative had it addressed how the testing of this conceptual model might inform other health literacy research and theory beyond the current population.

4. Approach:

Strengths

- Guidance of a conceptual model and appropriate hypotheses stemming from the model is a strength.
- Measurement is strong. Use of validated measures is appropriate.
- Analytic plan is well thought out.

Weaknesses

- The use of self-report to select participants may lead to misclassification and detract from the validity of the findings.
- The description of the translation work lacked detail about reliability and validity although previous work by the investigators lends credibility.
- The application would have been strengthened had the investigator provided a justification for the choice of the clinical scenarios. This seems untied to the argument that ER use is high among the deaf or the disparities that might be faced by the African American population or younger people that he is accessing.
- A plan for dissemination, particularly involving the community advisory board, would have strengthened the proposal.

5. Environment:

Strengths

- The research environment and collaboration at Michigan is excellent and compliments the ability of the investigators to accomplish the proposed work.
- The ties of the investigators across institutions are a huge plus.
- Involvement of a community advisory board is a strength.

Weaknesses

- None noted.

Protections for Human Subjects:

Acceptable Risks and/or Adequate Protections

- Respondent burden is high but addressed in the application.

Data and Safety Monitoring Plan (Applicable for Clinical Trials Only):

Not Applicable (No Clinical Trials)

Inclusion of Women, Minorities and Children:

- Sex/Gender: Distribution justified scientifically
- Race/Ethnicity: Distribution justified scientifically
- Inclusion/Exclusion of Children under 21: Including ages < 21 justified scientifically
- Includes individuals 18-21 years old and justified in proposal

Vertebrate Animals:

Not Applicable (No Vertebrate Animals)

Biohazards:

Not Applicable (No Biohazards)

Resubmission:

- The resubmission competently addresses the limitations in the 01 application

Resource Sharing Plans:

Unacceptable

- The direct cost budget exceeds $500,000 in any single year and the investigators have failed to provide a comprehensive data sharing plan as required.

Budget and Period of Support:

Recommend as Requested

- More detailed explanation of how the travel costs were established is appropriate
- The basis for consultant compensation is not described adequately

CRITIQUE 3

Significance: 1
Investigator(s): 1
Innovation: 1
Approach: 2
Environment: 1

Overall Impact: This resubmitted application proposes research focusing on health literacy in Deaf individuals. There is a substantial gap in the literature on health literacy in the deaf population which supports the significance of this proposed project. The investigator team is led by a highly qualified principal investigator who is deaf. Other members of the investigator team have the needed training and experience to carry out their roles on the project. The project will be implemented in four sites. The research plans for this application are complex and employ both quantitative and qualitative methods as well as novel technology. Overall this is a very strong application with enthusiasm diminished to a minor extent by the complexities that come with implementation across four sites.

1. Significance:
Strengths

- The application points out that the deaf population experiences social marginalization and health disparities association with challenges in health communications and limited literacy.
- The deaf population has knowledge gaps related to sexual health, cancer, preventive health and cardiovascular disease.

Weaknesses

- None noted

2. Investigator(s):
Strengths

- Dr. McKee, the proposed principal investigator has a strong record of achievement academically and in research. He lists 15 publications in print and in press since 2007. Of particular relevance to this application, he adapted and validated the Newest Vital Sign health literacy assessment tool for the ASL community. He is well qualified to lead this proposed project.
- Other investigators on the project have the needed training and experience to carry out their roles on the project.

Weaknesses

- None noted

3. Innovation:
Strengths

- There is a lack of research on health literacy in the deaf population. This proposed project is likely to provide results that will contribute to filling this gap in knowledge.
- The inclusion of technologist such as eye gaze and online search tracking support the novelty of this proposed project.

Weaknesses

- None noted

4. Approach:
Strengths

- The research methods section of the application is well organized and presented clearly. The proposed methods map well to the specific aims of the application.
- The preliminary research presented supports the application from a conceptual point of view as well as demonstrating the ability of the investigator team to carry out the proposed project.

- The mixed method approach to the project is well-reasoned. The plans to begin with a quantitative component followed by qualitative inquiry appears to a logical approach to increasing understanding of issues related to social marginalization and low health literacy.
- The previous review suggested that specific aim 3 plans were not clearly described. Plans for participant selection, data collection and analysis for aim 3 have been clarified in this resubmission.

Weaknesses

- The logistics of the project remain complex and while the investigator team is well qualified and institutional resources are adequate, there is concern regarding feasibility. Identification of contingency plans for participant recruitment and other aspects of the project would strengthen the application.

5. Environment:

Strengths

- The resources available to support the project are adequate.

Weaknesses

- None noted

<u>Protections for Human Subjects:</u>

Acceptable Risks and/or Adequate Protections

- Human subjects protections are acceptable

Data and Safety Monitoring Plan (Applicable for Clinical Trials Only):

Not Applicable (No Clinical Trials)

<u>Inclusion of Women, Minorities and Children:</u>

- Sex/Gender: Distribution justified scientifically
- Race/Ethnicity: Distribution justified scientifically
- Inclusion/Exclusion of Children under 21: Including ages < 21 justified scientifically
- Inclusions are acceptable

<u>Vertebrate Animals:</u>

Not Applicable (No Vertebrate Animals)

<u>Biohazards:</u>

Not Applicable (No Biohazards)

<u>Resubmission:</u>

- This revised application is responsive to the previous review. The revisions to the research plan have strengthened the application.

Budget and Period of Support:

Recommend as Requested

THE FOLLOWING SECTIONS WERE PREPARED BY THE SCIENTIFIC REVIEW OFFICER TO SUMMARIZE THE OUTCOME OF DISCUSSIONS OF THE REVIEW COMMITTEE, OR REVIEWER'S WRITTEN CRITIQUES, ON THE FOLLOWING ISSUES:

PROTECTION OF HUMAN SUBJECTS (Resume): ACCEPTABLE. No concerns.

DATA AND SAFETY MONITORING PLAN (Resume): ACCEPTABLE.

INCLUSION OF WOMEN PLAN (Resume): ACCEPTABLE.

INCLUSION OF MINORITIES PLAN (Resume): ACCEPTABLE.

INCLUSION OF CHILDREN PLAN (Resume): ACCEPTABLE.

COMMITTEE BUDGET RECOMMENDATIONS: Reviewers noted the following concerns regarding the proposed budget: 1) “More detailed explanation of how the travel costs were established is appropriate; 2) The basis for consultant compensation is not described adequately.”

SCIENTIFIC REVIEW OFFICER’S NOTES: The NIH special practice for new investigator R01 applications reviewed in the Center for Scientific Review study sections applies to this application. Resubmission (amended -A1) R01 applications from new investigators may be submitted on a special receipt date for review in the very next review cycle. See this notice in the NIH Guide for Grants and Contracts for more details: http://grants.nih.gov/grants/guide/notice-files/NOT-OD-11-057.html. You should contact the NIH program officer whose name is shown in the upper left hand corner of page one of this Summary Statement for information about whether this application may be fundable or whether you will need to submit an amended application. The program officer can also help you decide whether the changes and improvements necessary to address the weaknesses noted in the reviewers' critiques could be accomplished in the relatively short time available. You are also strongly advised to seek input from mentors, your Department chair, etc. If you choose to submit a resubmission application for the next review cycle under this policy for new investigators, your amended application must be received at NIH no later than Wednesday, December 10, 2014. You may, of course, choose to take more time to resubmit your application. If so, you should prepare the resubmission for the normal dates for amended applications as specified in this table: http://grants1.nih.gov/grants/funding/submissionschedule.htm.

NIH has modified its policy regarding the receipt of resubmissions (amended applications). See Guide Notice NOT-OD-14-074 at http://grants.nih.gov/grants/guide/notice-files/NOT-OD-14-074.html. The impact/priority score is calculated after discussion of an application by averaging the overall scores (1-9) given by all voting reviewers on the committee and multiplying by 10. The criterion scores are submitted prior to the meeting by the individual

reviewers assigned to an application, and are not discussed specifically at the review meeting or calculated into the overall impact score. Some applications also receive a percentile ranking. For details on the review process, see http://grants.nih.gov/grants/peer_review_process.htm#scoring.

MEETING ROSTER

Center for Scientific Review Special Emphasis Panel
CENTER FOR SCIENTIFIC REVIEW
PAR13-132: Understanding and Promoting Health Literacy
ZRG1 HDM-X (56) R
May 08, 2015

CHAIRPERSON

BELGRAVE, LINDA LISKA, PHD
ASSOCIATE PROFESSOR
DEPARTMENT OF SOCIOLOGY
UNIVERSITY OF MIAMI
CORAL GABLES, FL 33146

MEMBERS

AMMERMAN, ALICE S, DRPH
PROFESSOR
CENTER FOR HEALTH PROMOTION
AND DISEASE PREVENTION
GILLINGS SCHOOL OF PUBLIC HEALTH
UNIVERSITY OF NORTH CAROLINA AT CHAPEL HILL
CHAPEL HILL, NC 27599

CAMPO, MICHELLE LYNN, PHD
ASSOCIATE PROFESSOR
DEPARTMENT OF COMMUNITY
AND BEHAVIORAL HEALTH
UNIVERSITY OF IOWA
IOWA CITY , IA 52242

DAVE, JAYNA MARKAND, PHD
ASSISTANT PROFESSOR
BAYLOR COLLEGE OF MEDICINE
SUGAR LAND, TX 77498

DIGNAN, MARK B, PHD
PROFESSOR
DEPARTMENT OF INTERNAL MEDICINE
PREVENTION RESEARCH CENTER
MARKEY CANCER CENTER
UNIVERSITY OF KENTUCKY
LEXINGTON , KY 40536

LIU, GILBERT C, MD
ASSOCIATE PROFESSOR AND DIRECTOR
DEPARTMENT OF PEDIATRICS
UNIVERSITY OF LOUISVILLE
LOUISVILLE, KY 40202

MADIGAN, ELIZABETH ANN, RN, PHD, BSN
ASSOCIATE DEAN FOR ACADEMIC AFFAIRS
INDEPENDENCE FOUNDATION PROFESSOR
BOLTON SCHOOL OF NURSING
CASE WESTERN RESERVE UNIVERSITY
CLEVELAND, OH 44106

MASTIN, TERESA
PROFESSOR OF PUBLIC RELATIONS AND ADVERTISING
(PRAD)
COLLEGE OF COMMUNICATION
DEPAUL UNIVERSITY
CHICAGO, IL 60604

MILGROM, PETER M, DDS
PROFESSOR
SCHOOL OF DENTISTRY
UNIVERSITY OF WASHINGTON
SEATTLE, WA 981957475

SHAYA, FADIA TOHME, PHD
VICE-CHAIR FOR ACADEMIC AFFIARS AND DIRECTOR
OF RESEARCH
PHARMACEUTICAL HEALTH SERVICES RESEARCH
CENTER ON DRUGS AND PUBLIC POLICY
SCHOOL OF PHARMACY
UNIVERSITY OF MARYLAND
BALTIMORE, MD 21201

VEINOT, TIFFANY CHRISTINE ELIZABETH, PHD
ASSOCIATE PROFESSOR
SCHOOL OF INFORMATION
UNIVESITY OF MICHIGAN
ANN ARBOR, MI 481091285

WETHINGTON, ELAINE , PHD
PROFESSOR
DEPARTMENT OF HUMAN
DEVELOPMENT AND SOCIOLOGY
CORNELL UNIVERSITY
ITHACA, NY 14853

MAIL REVIEWER(S)

LAMNERT, WILLIAM E, PHD
ASSOCIATE PROFESSOR
DEPT OF PUBLIC HEALTH AND PREVENTIVE MEDICINE
OREGON HEALTH & SCIENCE UNIVERSITY
PORTLAND, OR 97239

SCIENTIFIC REVIEW OFFICER

HENRY, REBECCA , PHD
SCIENTIFIC REVIEW OFFICER
CENTER FOR SCIENTIFIC REVIEW
NATIONAL INSTITUTES OF HEALTH
BETHESDA, MD 20892

EXTRAMURAL SUPPORT ASSISTANT

NJOKU, PHILIP C
CENTER FOR SCIENTIFIC REVIEW
NATIONAL INSTITUTES OF HEALTH
BETHESDA, MD 20892

Consultants are required to absent themselves from the room during the review of any application if their presence would constitute or appear to constitute a conflict of interest.
